# Research on the Operating Mechanism of E-Commerce Poverty Alleviation in Agricultural Cooperatives: An Actor Network Theory Perspective

**DOI:** 10.3389/fpsyg.2022.847902

**Published:** 2022-04-13

**Authors:** Na Xu, Chi Xu, Yuanbo Jin, Zhenjie Yu

**Affiliations:** ^1^School of Economics and Trade Management, Wenzhou Vocational College of Science and Technology, Wenzhou, China; ^2^Information Construction and Management Center, Wenzhou Vocational College of Science and Technology, Wenzhou, China; ^3^Office of Academic Research, Wenzhou Vocational College of Science and Technology, Wenzhou, China

**Keywords:** E-commerce poverty alleviation, farmer cooperative, actor network theory, poverty eradication, targeted poverty alleviation, sustainable development goals, UN SDGs

## Abstract

E-commerce poverty alleviation has become a new wisdom in China’s rural poverty alleviation, but there are a few empirical researches on e-commerce poverty alleviation based on farmer cooperatives. Taking four typical poverty counties in Zhejiang Province as an example, based on the actor network theory (ANT), this paper defines the participants and their obligatory passage point (OPP) from the e-commerce poverty alleviation actor network (EPAAN), combs the roles and interest demands of various stakeholders, and constructs the EPAAN model based on farmer cooperatives according to the translation process. We found that the EPAAN is a heterogeneous network by constantly updating, which consists of human actors with cooperatives as the main body and non-human actors. Moreover, our study illustrates that the formation and operation mechanism of cooperative e-commerce poverty alleviation network alliance under the background of targeted poverty alleviation can be well analyzed with the help of actor network theory. This study contributes to provide a feasible Chinese plan for the cause of poverty eradication all over the world, and provides a great reference value for global poverty governance.

## Introduction

Poverty has become one of the most serious problems faced by the whole world. According to the Global Multidimensional Poverty Index 2020 report released by the United Nations, a total of 1.3 billion people in the world are in a “multidimensional poverty state” now, and there are great differences in the degree of poverty among countries and regions within countries (Oxford Poverty and Human Development Initiative, 2020). Moreover, the first item of the United Nations’ 2030 Sustainable Development Goals points out that all forms of poverty will be eliminated on a global scale in 2030 (Peng and Li, 2017). Poverty eradication is the common task of humankind. China has always paid great attention to poverty alleviation and implemented a series of poverty alleviation national plans for medium and long term development. From relief-based poverty alleviation to development-driven poverty alleviation and then to targeted poverty alleviation, it has made historic achievements in poverty alleviation in China. According to the white paper titled “Poverty Alleviation: China’s Experience and Contribution” issued by China’s State Council Information Office in April 2021, it showed that 770 million people in rural areas all shook off poverty by the current poverty threshold after reform and opening up. By the international poverty standard of the World Bank, the China’s poverty reduction population accounted for more than 70% of the global poverty reduction population in the same period. At the end of 2020, China achieved the goal of eliminating extreme poverty on time. The 98.99 million people in rural areas that were living below the current poverty threshold all shook off poverty, all the 128,000 impoverished villages and 832 designated poor counties got rid of poverty. China has eliminated poverty over entire regions and eradicated extreme poverty (The State Council Information Office of the People’s Republic of China, 2021). Consequently, China which accounts for nearly one fifth of the world’s population has comprehensively eradicated extreme poverty. It has realized 10 years ahead of schedule to achieve the poverty alleviation goal of the United Nations 2030 agenda for sustainable development.

Since the strategy of targeted poverty alleviation is implemented in China, the rural impoverished population has decreased significantly. It has made a remarkable achievement and made a great contribution to the cause of global poverty alleviation. As a new thing under the targeted poverty alleviation strategy, e-commerce poverty alleviation makes a deep integration with industrial poverty alleviation by taking e-commerce as the carrier. It has become an important channel among the poverty alleviation work in China. In 2015, the Poverty Alleviation Office of the State Council listed the “E-commerce Poverty Alleviation Project” as one of the ten targeted poverty alleviation projects (Wei, 2017; Wang and Peng, 2020). In November 2016, the file titled “Guidelines on Promoting Targeted Poverty Alleviation through E-commerce” was jointly issued by the Poverty Alleviation Office of the State Council and other 16 ministries and commissions of the CPC Central Committee. The guidelines pointed out that the resources from all parties should be coordinated to jointly support and promote e-commerce targeted poverty alleviation (The National Rural Revitalization Administration, 2016). In December 2018, the notice on further highlighting the poverty alleviation orientation and making every effort to implement the e-commerce poverty alleviation policies was issued by the Ministry of Commerce. From the notice, based on the resource endowment and industrial characteristics, it should develop diversified e-commerce supply chains such as subsidiary agricultural products, tourism, catering and folk customs according to local conditions, improve the interest connection mechanism, and explore ways to achieve targeted assistance to poor households (Ministry of Commerce People’s Republic Of China, 2018). In June 2020, the notice on doing a good comprehensive work for e-commerce in rural areas in 2020 was jointly issued by the Ministry of Finance and other 3 ministries and commissions of the CPC Central Committee. It clearly stated that using e-commerce can broaden the sales channels of agricultural and livestock products, overcome the impact of the epidemic and help increase income and get rid of poverty (Zhang, 2020). These policies highly promote the development of rural e-commerce and e-commerce poverty alleviation in poor areas based on different perspectives. Moreover, from the practice of e-commerce poverty alleviation in recent years, e-commerce poverty alleviation has indeed achieved remarkable effect in poor areas, especially based on cooperatives which are with farmers as the main body. Therefore, in this paper, we focus on e-commerce poverty alleviation based on cooperatives, and attempt to address such a research question as “how cooperatives lead e-commerce poverty alleviation to play an important role in poverty-stricken areas, ensure that e-commerce poverty alleviation benefits all participants, and form a dynamic operation mechanism.”

The design and implementation of our research are under the guidance of actor network theory (Law, 1999). It is a kind of sociological method that interweaves various heterogeneous actors to build a network, and solves a specific problem through the network development (Law, 1999). The adoption of ANT is helpful to comprehensively sort out the relationship of interdependence, interaction and transformation between individuals and systems in the process of e-commerce poverty alleviation based on cooperatives. Based on the perspective of ANT, this paper draws on the experience of ANT theoretical framework, and provides a new research method for studying the development process of e-commerce poverty alleviation based on cooperatives. Through field research and case analysis, this paper attempts to translate and analyze the practice process of e-commerce poverty alleviation based on cooperatives in China, so as to find existing problems and countermeasures. In order to promote poverty alleviation in China and other developing countries for e-commerce poverty alleviation, it will put forward some useful theoretical value and practical significance.

The rest of this paper is structured as follows. In the second part, we review the relevant literature. In the third part, we describe the research method, sample strategy and data collection. In the fourth and fifth parts, we will respectively state our findings and a discussion. It will conclude in the sixth part.

## Literature Review

In this section, it mainly includes 4 parts. We summarize the role and influence of e-commerce in poverty alleviation in the first part. The second part is to explore the influence of farmer cooperatives in the process of poverty alleviation. We summarize the ANT framework and explore its research in related areas of poverty alleviation in the third part. The last part is to discuss the innovation of this study.

### E-Commerce Toward Poverty Alleviation

Nowadays, with the continuous popularization of e-commerce, people have realized the importance of e-commerce, and e-commerce has become an important engine for the development of the national economy. In the whole poverty alleviation system, e-commerce poverty alleviation has gathered more and more consensus, and it is becoming a new channel for poverty alleviation and a new highlight in the development of e-commerce. From some foreign researches, foreign scholars are paying more and more attention to the role of information and communication technology and e-commerce in anti-poverty, and have reached the same consensus on their role in poverty alleviation. For example, by studying the relationship between information technology and anti-poverty, it is considered that information technology plays a positive role in anti-poverty (Becerril and Christian, 2020). Moreover, the application of information and communication technology in rural areas plays an important role in increasing farmers’ income and promoting rural economic development (Raza et al., 2020; Bjorn et al., 2021). It can establish the foundation for the formation of rural e-commerce ecosystem and contribute to the self-development of rural e-commerce (Hsu et al., 2020). Simultaneously, the usage of e-commerce can help farmers in developing countries play a positive role in expanding the sales channels of agricultural products and reducing transaction costs and transaction risks (Dsouza, 2014; Liu et al., 2020; Chen et al., 2021). It is conducive to accelerating the process of agricultural industrialization and expanding income. From some domestic researches, domestic scholars mainly focus on the e-commerce poverty alleviation’s conception, development models, paths and countermeasures. The conception of “e-commerce poverty alleviation” was first proposed by Wang and Wang (2015), who summarized the basic concept, forms, necessity, function incentives and implementation measures of e-commerce poverty alleviation (Wang and Wang, 2015; Yao, 2019). So far, there is no final definition of e-commerce poverty alleviation, but the thought is gradually clear. Scholars have fully discussed its connotation and formed a relatively consistent view on e-commerce poverty alleviation, which is that using e-commerce promotes families in poor areas to get rid of poverty and promote the development of local industries (Zhou, 2015; Wang, 2017; Wei, 2018; Lin and Wang, 2020; Zhang, 2021). With the development of e-commerce poverty alleviation in poor areas in China, some scholars investigated the current situation of characteristic industries and agricultural products markets in poor areas, and explored the main models and countermeasures of e-commerce poverty alleviation suitable for poor areas (Zhang and Wang, 2016; Deng and Liu, 2021; Gao et al., 2021). There are some typical representatives of e-commerce poverty alleviation models such as “Longnan Model,” “Dangshan Model,” “Chenxian Model,” “Qingchuan Model,” “Tongyu Model,” “Shaji Model,” and so on (Research Group for Longnan’ Poverty Alleviation via E-commerce, 2018; Dong et al., 2019; Guo and Mi, 2019; Zan and Wang, 2020; Chen, 2021). Some scholars analyzed the main ways of rural e-commerce poverty alleviation in combination with local practice, such as increasing income, saving expenditure and improving energy, and so on (Lin, 2016; Yan et al., 2018; Zhang, 2018). According to the existing main problems, they put forward some countermeasures and suggestions in infrastructure, talent training, policy support, resource docking and so on (Wei, 2017; Yi, 2018; Wang, 2020; Gong et al., 2021).

### Farmer Cooperatives Toward Poverty Alleviation

Because of the low degree of farmers’ organization (Ao et al., 2021), the weak ability of connecting the large market and resisting risks, it is difficult for poor farmers to continuously increase their income. So it has become one of the most prominent problems that poor farmers are returning to poverty after getting rid of poverty. Therefore, the most effective way is to improve the level of farmers’ organization, which can better solve the problem of farmers’ poverty. The farmer cooperatives are the main form of improving the degree of farmers’ organization. They play an important role in promoting farmers’ income, responding to market competition, and improving the level of agricultural organization and standardization. In China, government always pays high attention to the development of cooperatives. In October 2006, the “law of the People’s Republic of China on Specialized Farmer Cooperatives” was promulgated, and it was revised in December 2017 and implemented in July 2018. During those years, it was issued a series of laws, regulations and supporting policies according to cooperatives in China. Looking at foreign studies, foreign scholars more agree that cooperatives play a role in poverty alleviation, and pay more attention to the research on the role of cooperatives, the impact on poverty alleviation and increasing farmers’ income. For example, small-scale farmers are often unable to bear high transaction costs, but cooperatives can take advantage of economies of scale when providing services (Xu, 2018; Arunrat et al., 2021),they can facilitate small farmers to enter differentiated markets, reduce transaction costs (Peng and Fu, 2018), and help small farmers overcome information deficiencies related to production technology and market access (An et al., 2015). Getnet and Anullo (2012) analyzed data from Ethiopia and believed that agricultural cooperatives are important to support the development of people’s livelihood and poverty alleviation, and play a role in saving costs, increasing income and saving in the production and life of farmers. Onyilo and Adong (2019) emphasized the important impact of cooperatives on farmers’ poverty alleviation by exploring the marketing and credit policy reform of agricultural cooperatives in Uganda. Gava et al. (2021) studied the effects of Bosnia and Herzegovina farmer cooperatives in reducing rural poverty and preventing poverty. Looking at domestic studies, domestic scholars currently focus on the mechanism, model and countermeasures of cooperatives in targeted poverty alleviation. For example, some scholars agree with the functional value and mechanism of cooperatives very much in promoting industrial targeted poverty alleviation (Bai et al., 2017; Shao and Yu, 2017; Feng et al., 2020; Zhou et al., 2020). Other scholars have explored the poverty alleviation model of cooperatives from different viewpoints, such as the “farmer cooperative+” industrialized targeted poverty alleviation model (Liao et al., 2016), the “government-market-community-cooperative” four-in-one poverty alleviation model (Li and Chen, 2017), and the collaborative poverty alleviation model of rural collective economic organizations and cooperatives (Ding, 2020), and so on. Moreover, some scholars have discussed the problems of cooperatives in assisting targeted poverty alleviation, and put forward countermeasures and suggestions to improve the effectiveness of cooperatives in poverty alleviation (Bai and Li, 2017; Guo and Zhao, 2018; Yuan et al., 2021).

### Actor Network Theory Framework

Actor network theory (ANT) was founded by Latour, Callon, and Law in the middle of 1980s,who were famous scholars belonged to Sociology of Scientific Knowledge in French (Law, 1999). Callon was the first to propose three concepts, namely the actor network, actor, translation (Callon, 1984). Law followed the research method of Callon and put forward how to maintain the stability of actor network (Law, 1984). Based on the theoretical research of Callon and Law, Latour (1987) further enriched the theoretical connotation of actor network, and put forward that its broad definition meant that a scientific activity was completed by the joint participation of “actors” in different roles. In order to realize the benefits conferred by participating in the activity, various actors play different roles or functions, thus forming an inseparable network, and the ultimate interests of actors are realized through the joint linkage between themselves and other actors. At the same time, the core concepts of “actor,” “actor network,” “obligatory passage point,” and “translation” are described in more detail, and their theories are applied to scientific practice (Latour, 1997).

**FIGURE 1:**
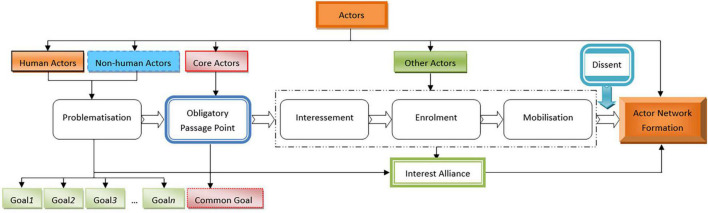
The framework of actor network theory (ANT).

Actor network theory is based on the principle of generalized symmetry, which gives all actors equal status (Callon, 1984). Here “actor” refers to any element that plays a role in the process of scientific practice and research. It includes both animate human actors and inanimate non-human actors. Human actors include individuals, groups, organizations, institutions, etc. Non-human actors include technology, equipment, software, platforms, communication hardware, infrastructure standards, etc. (Walsham, 1997). The “actors” can be divided into core actors and other actors. The actors who play a leading role in the construction of the actor network can be called core actors (Luo, 2013). The concept of “actor” breaks the distinction between “human” and “non-human,” and eliminates the dual opposition between subject and object, nature and society (Latour, 1987), which has profound revolutionary significance. An “actor network” refers to a constant dynamic and non-transcendental network formed by closely connecting all actors as “network nodes” around a common goal by defining and endowing their interests, roles, functions and status (Latour, 1987). “Translation” means that core actors translate and explain the language, problems, identity and interests of other actors in their own words, bring them into the network, and combine the interests of various actors to form a solid network interest alliance (Latour, 1987). The translation process includes problematization, interessement, enrolment, and mobilization (Callon, 1986). In order to enable all actors to reach a consensus, take unified action and ensure the smooth operation of the whole translation process, it is necessary to pass through the “obligatory passage point” (OPP). OPP is a scheme proposed by the initiator of the actor network, which solves the problems faced by other actors, achieves their own goals, and highly unifies the thoughts and behaviors of all actors according to their own purpose. OPP is a necessary point for all actors going through. The actors only pass through OPP, then they can be allowed to enter actor network. Moreover, their satisfaction with problem solving directly affects the stability of the network alliance (Latour, 1997; Liu et al., 2016). The framework of ANT is shown in Figure 1 as follows.

Since the birth of ANT, it has been highly paid attention by academic circles at home and abroad. Now ANT has been widely used in many fields of social sciences. For example, some foreign scholars such as Voeten et al. (2015), Saba et al. (2018); Hagberg and Fuentes (2018), and some domestic scholars such as Liu (2011); Huang (2016), Wang et al. (2020) they respectively introduce ANT in different areas such as poverty alleviation, farmer cooperatives, and e-commerce. Taking ANT as the foundation of endogenous development, scholars explore the composition of various interest groups, the relationship among various stakeholders and the construction of dynamic network, and put forward corresponding improvement countermeasures.

### The Innovation of E-Commerce Poverty Alleviation Actor Network Based on Cooperatives From the Perspective of Actor Network Theory

The application of e-commerce to poverty alleviation is an important government initiative in China. As this practical activity has not been carried out for a long time, there are many problems about e-commerce in the process of cooperatives poverty alleviation, such as multiple actors, cooperation mechanism and benefit distribution. Throughout the existing studies, scholars at home and abroad have adopted different research methods to analyze the theories of e-commerce poverty alleviation, cooperatives and ANT from different perspectives. It has reference significance and lays a research foundation for the development of e-commerce poverty alleviation based on cooperatives.

Actor network theory is an important theoretical tool for analyzing multi-agent relationships, complex and dynamic system networks. Using the theoretic framework of ANT to e-commerce poverty alleviation and cooperatives, it can construct the model of e-commerce poverty alleviation actor network (EPAAN) based on cooperatives. The EPAAN model can more comprehensively and systematically analyze the interaction among various actors in the implementation of e-commerce poverty alleviation in cooperatives, and effectively solve the problems in the process of their cooperation mechanism and the rational distribution of interests and responsibilities. It is conducive to the analysis and construction of the operating mechanism of e-commerce poverty alleviation in the implementation of cooperatives. Therefore, it is fully feasible to apply ANT to the research on e-commerce poverty alleviation based on cooperatives. Based on previous studies, it is hypothesized that cooperatives in the EPAAN may have a positive impact on farmers in poverty-stricken areas to get rid of poverty and become rich. This hypothesis will be tested and evaluated in the current study.

In summary, the research on the development of e-commerce poverty alleviation based on cooperatives from the perspective of ANT provides a new idea for relevant research. It is different from traditional research that tends to focus on business management, Internet technology, information and communication technology, etc. And it breaks the limitations of research perspectives. Simultaneously, it conducts research from the perspective of influential actors in the entire e-commerce poverty alleviation network, fully explores the relationship among various elements in the construction of the e-commerce poverty alleviation network, and interprets the construction process of EPAAN from a systematic and comprehensive perspective, and proposes the dynamic and sustainable development strategies. To some extent, this study fills the research gap on the role of cooperatives in e-commerce poverty alleviation and exploring the internal relationship of various actors in the EPAAN from the perspective of ANT.

## Research Design

### Methodology

Since the scholar Wang and Wang (2015) put forward the concept of “e-commerce poverty alleviation” in China, e-commerce poverty alleviation has been widely concerned and discussed by scholars from all walks of life on society. Because the main battlefield of e-commerce for poverty alleviation is in rural areas in China, especially in poor rural areas, it is difficult to obtain data through broad surveying methods. Therefore, we adopt a qualitative, multi-case study research design (Collis and Hussey, 2003; Yin, 2003). For a specific phenomenon, e-commerce poverty alleviation as a new thing involves many bodies, we adopt the maximum variation sampling strategy as a research strategy (Eisenhardt, 1989; Voss et al., 2002). It can deeply explain and analyze all its variations, and explore its law of development (Creswell, 1998; Merriam, 2009; Freitas et al., 2018). Now rural e-commerce has fully exploded. In particular, Zhejiang Province has played a leading role in the national e-commerce poverty alleviation practice. Through the e-commerce poverty alleviation based on cooperatives, it has brought tangible changes to more and more impoverished counties, impoverished villages, and impoverished households. Hence, we aim to build an EPAAN model based on cooperatives, and explore how multiple entities can form joint forces in the e-commerce poverty alleviation process to help targeted poverty alleviation. Its operation mechanism will be explored in our case. In our research, the case study mainly follows “theory-practice-theory.” Firstly, we combine ANT to form perceptual knowledge on the basis of preliminary investigations, and construct a preliminary theoretical model through theoretical derivation. Secondly, a large amount of data is collected from several counties in Zhejiang that were once underdeveloped, and the preliminary theoretical model is continuously tested, revised and improved through data analysis, so that the theoretical model is continuously enriched and improved, until it finally reaches theoretical saturation. Moreover, the final theory can effectively explain the phenomenon of e-commerce poverty alleviation based on cooperatives. Finally, we discuss the practical significance of the final theoretical model.

### Case Introduction

As a developed province in the eastern coast of China, Zhejiang is the first region to completely eliminate absolute poverty in China (The State Council Information Office of the People’s Republic of China, 2015). However, due to different natural endowments, regional development is not balanced. Especially the economic development of counties and townships in southwestern Zhejiang is relatively backward, and there are some poor counties and poor people (The State Council Information Office of the People’s Republic of China, 2015). Therefore, our study selects Wencheng county, Taishun county, Yongjia county, and Suichang county in the southwestern Zhejiang Province as the research sites. Their common feature is that they once belonged to the 26 underdeveloped counties in Zhejiang Province (Pan, 2015), and the mountainous area of each county is relatively large, which is the epitome of “seven mountains, two rivers and one field” in Zhejiang Province. Therefore, how to develop the “green water and green mountains” of the underdeveloped counties into “golden mountains and silver mountains,” e-commerce has a great promising. On the one hand, the government of Zhejiang Provincial has increased investment in infrastructure construction, and on the other hand, it has helped these counties actively explore e-commerce and connect with local characteristic industries. For example, it has helped local agricultural products walk into thousands of households across the country through e-commerce, and has made some achievements in helping farmers increase their income. Due to effective measures, these counties have achieved obvious poverty alleviation effects. In August 2020, the general office of Zhejiang Provincial Party Committee announced the development performance evaluation results of 26 counties including Chun’an in 2019. Among them, Taishun county, Wencheng county, and Yongjia county ranked among the top three (Xu, 2020) in Zhejiang Province. However, e-commerce plays an important role in this poverty alleviation project. For a long time, the experience of poverty alleviation in Zhejiang has been at the forefront in China. This paper studies the e-commerce poverty alleviation problems of once underdeveloped counties in developed provinces. It can provide a useful reference and practical significance for governments at all levels in different regions of economic development to formulate e-commerce poverty alleviation policies. Meanwhile, it has played a good complement and perfect significance for the e-commerce poverty alleviation theory in the developing countries.

### Data Collection

In our study, all data collection is obtained by site visits, face-to-face semi-structured interviews, and archival data from the internet from September 2019 to January 2021. According to the feasibility of e-commerce in cooperatives in counties and the actors involved in the process of poverty alleviation, the interviewees are determined as cooperative principals, farmers (including ordinary farmers and poor farmers), grass-roots cadres/government officials, agriculture-related e-commerce enterprises, logistics enterprises, experts from universities and research institutes, e-commerce elites, consumers, etc.

Through the website of Zhejiang Provincial Department of Agriculture and Rural Affairs, we initially contacted 42 provincial-level demonstration farmer cooperatives with practical representatives from four research sites, and then selected 20 cooperatives that met our research requirements. In the field investigation, we carried out focus-group interview and made observations. After interviewing 20 cooperative principals, we purposefully selected 40 farmer representatives (e.g., benefits economic distribution from e-commerce poverty alleviation) and 12 managers of agriculture-related e-commerce enterprises (e.g., a minimum of 5 years of experience in e-commerce sales of agricultural products with cooperatives), 12 managers of logistics enterprises (e.g., a minimum of 5 years of experience in providing logistics services for cooperatives), and 8 village secretaries (e.g., a minimum of 3 years of experience in poverty alleviation in the village). In the first round of data collection, we focused on collecting data to comprehensively understand the operation of e-commerce poverty alleviation based on cooperatives in each research site. In the second stage of data collection, we collected data to focus on understanding the effectiveness of e-commerce poverty alleviation for all participants.

In order to ensure the validity and reliability of the construct, we interviewed 6 agricultural experts (e.g., a minimum of 12 years of experience in guiding grass-roots agricultural industry) and 6 backbone teachers of e-commerce (e.g., a minimum of 8 years of e-commerce poverty alleviation experience) from two agricultural colleges and universities. Moreover, we interviewed 28 consumers who had experience in purchasing agricultural products by online shopping. Hence, it increased the interview sample to 132 persons. It is shown in Table 1.

**TABLE 1:** Interview sample structure.

Geographical position	Sample category	Sample size
	Farmer cooperative	20
Wencheng county	Cooperative principal	20
Taishun county	Farmer	40
Yongjia county	Village secretary	8
Suichang county	Manager of agricultural e-commerce enterprise	12
	Manager of logistics enterprise	12
Zhejiang province	Agricultural expert	6
	Backbone teacher of e-commerce	6
	Consumer	28

Through field investigation and in-depth interviews, we centered around the case location to understand their regional characteristics, geographical environment, characteristic industries, cooperative participation, farmer participation, regional culture, folk customs, rural e-commerce and e-commerce poverty alleviation development characteristics. Meanwhile, we understood the interactions of different actors such as government, cooperatives, farmers, agriculture-related e-commerce companies, and logistics companies. During the interview, we will do some fine-tuning for the interview outline according to the category of interviewees, but we still insisted that the interview goal is “the influence and role of e-commerce poverty alleviation in the operation of cooperatives” as the core content. The duration of each interview will be 60–120 min. According to the data collection method (Yin, 2013), we will convert all interview data into text materials, and perform coding and descriptive statistical analysis for the relevant data, and some interviewees will accept more than once interview.

Finally, during our data analysis stage, as suggested by Yin (2009), we should pay much attention to establishing all chains of evidence that can trace back from the initial research questions to the final case study conclusions in our case study. Once any evidence source is found incomplete or missing in the process of system analysis, we will be more active in collecting and updating more data to track until we find the origin of the problem. It will be used to either substantiate or disconfirm our theoretical.

## Findings

### An Integrated Evolution Model of E-Commerce Poverty Alleviation Actor Network

Through theoretical deduction and case analysis, our key findings are based on the development process of EPAAN based on cooperatives, the composition of many actors and the implementation process of e-commerce poverty alleviation network. During our field investigation, we found that Suichang county had earlier come into contact with e-commerce than other counties. Combined with the local characteristic agriculture in the mountainous area, Suichang county sold characteristic agricultural products such as bamboo charcoal, camellia oil and tea through the e-commerce platform, and formed an e-commerce poverty alleviation network to drive a large number of poor farmers to increase their income. Therefore, through the comparison of relevant sample data in four counties, it is found that the cooperatives from Suichang county have done better in developing the e-commerce poverty alleviation network based on cooperatives than those in other counties. During our site visits, take an interview question of “please describe whether e-commerce poverty alleviation can have a positive impact on cooperatives and its specific performance” as an example, the 20 interviewed cooperatives compared the traditional market-based poverty alleviation with the current use of e-commerce poverty alleviation, and unanimously agreed that e-commerce poverty alleviation can help greatly increase the sales of agricultural products and increase the income of cooperatives. For example, on the question of “please describe whether cooperatives participating in e-commerce poverty alleviation can have a positive impact on farmers and its specific performance,” the interviewed farmers agreed with joining cooperatives and participating in e-commerce poverty alleviation. They all said that it can more effectively solve farmers’ problems on selling agricultural products than individual retailing in the traditional marketing way. It can greatly increase their income and achieve targeted poverty alleviation.

**FIGURE 2:**
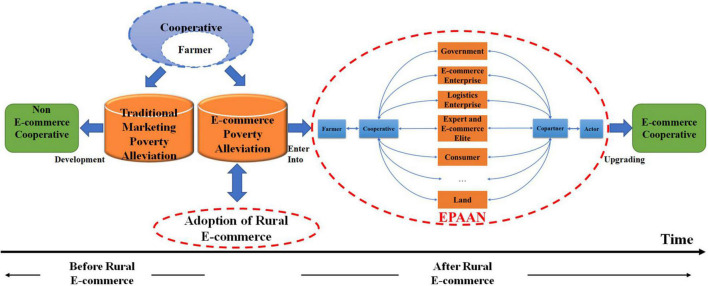
An integrated evolution model: E-commerce poverty alleviation actor network (EPAAN) based on cooperatives.

As noted above, on the development process of EPAAN based on cooperatives, we summarize our findings as an integrated evolution model shown in Figure 2. Before the emergence of e-commerce poverty alleviation, cooperatives used traditional marketing poverty alleviation to sell agricultural products to help poor farmers. However, with the development of e-commerce, cooperatives found that they could obtain a broader sales market and stronger bargaining power with the help of e-commerce. Hence, they began to use rural e-commerce for online sales of agricultural products, cooperated with other partners to establish an EPAAN around the common goal, and formed a solid interest alliance network.

In our model, it should pay attention to exert the subject consciousness of cooperatives, stimulate and mobilize the endogenous motivation of farmers through cooperatives to form the bottom-up targeted poverty alleviation. Cooperatives will gradually upgrade into e-commerce cooperatives in this model, and promote the continuous and steady increase in the income of poor farmers. Therefore, how to give full play to the main role of cooperatives in the process of e-commerce poverty alleviation, and how to ensure that stakeholders realize efficient alliances in the process of e-commerce poverty alleviation are key issues that need to be resolved urgently in the integrated development of e-commerce poverty alleviation and cooperatives. However, the translation process is the core content of the actor network. With the help of the translation process, the core actors can be closely linked with other actors around their common goal to form a network of interest alliance, which can effectively solve the above problems encountered by the EPAAN based on cooperatives.

### Actors Identification of E-Commerce Poverty Alleviation Actor Network

In order to fully understand the main body of the e-commerce poverty alleviation network system based on cooperatives, it is found that it contains many actors by sorting out the interview data and extracting relevant elements. According to the ANT, we can divide actors into human actors and non-human actors according to the first level classification. According to the second level classification, human actors can be divided into organization and individual, and non-human actors can be divided into material category and consciousness category. Therefore, the EPAAN includes human actors represented by organizations such as governments, cooperatives, enterprises, etc., as well as individual representatives such as leaders of relevant government departments, cooperatives principals, enterprises managers, experts and scholars, e-commerce elites, farmers, etc. Moreover, EPAAN also contains non-human actors represented by material categories such as land, funds and agricultural products, knowledge and technology, etc., as well as consciousness categories such as policies and regulations experience accumulation, etc. It is shown in Table 2.

**TABLE 2:** The actors composition of e-commerce poverty alleviation actor network (EPAAN).

Actor	Classification	Representative
Human actor	Organization	Governments, cooperatives, agriculture-related e-commerce companies, third party e-commerce platform enterprise, logistics companies, etc.
	Individual	Government officials, cooperative members, farmers, e-commerce workers, logistics workers, managers, technicians, consumers, etc.
Non-human actor	Material category	Land, funds, equipment, platforms, software, agricultural products, etc.
	Consciousness category	Thoughts, knowledge, experience, technology, system, social environment, policies and regulations, values, knowledge and skills, etc.

From the interview data, the realization of “e-commerce poverty alleviation” is a very complicated project. It needs not only the support of the government, cooperatives, agriculture-related e-commerce enterprises and so on, but also the support of land, policies, agricultural products, etc. Just as the ANT advocates, the EPAAN can be regarded as a place where human actors and non-human actors work and interact together. It does not give priority and key status to the power of either party. Driven by the common interests, these human actors and non-human actors cooperate and translate in different ways of action, and promote the formation and development of “e-commerce poverty alleviation” with cooperatives as the carrier.

### Implementation Process of E-Commerce Poverty Alleviation Actor Network

The implementation process of e-commerce poverty alleviation based on cooperatives is a process of continuous establishment and renewal of heterogeneous networks. Heterogeneous actors are accompanied by the change in the pattern of interests, and different actors will enter and exit the network at different stages. According to the ANT, the translation process of EPAAN mainly includes five stages: problematization, interessement, enrolment, mobilization and dissent. Combined with the content of the interview, we analyzed the five stages of the translation of EPAAN. We identified the core actors and OPP, analyzed the obstacles and benefits involved by different actors, and conducted consultations through core actors to find out the countermeasures to overcome the obstacles of various actors. Finally, it formed a stable and dynamic interests alliance of EPAAN.

#### Problematization and Obligatory Passage Point

Problematization is the initial stage of interest alliance of EPAAN. The core actors point out the ways for different actors to achieve their goals, and clarify the obstacles and problems faced by different actors for achieving their goals. Through the core actors, it finds out the OPP that all actors recognize and have to travel. The OPP connects the core actors and various heterogeneous actors into an actor network. Core actors are followers of network construction, and various heterogeneous actors cooperate with each other to achieve common goals. At present, farmer cooperatives are the fastest growing and most widely covered rural cooperative organizations in China. Therefore, cooperatives should be introduced into China’s e-commerce targeted poverty alleviation project and become core actors. They can use their authority and leadership to accurately identify, evaluate and assist poor farmers from the perspective of accuracy. According to the government’s poverty alleviation policies and resources, it can make use of the advantages of cooperatives to drive small farmers to connect to the big market, and empower the participants of rural e-commerce through e-commerce, so that the poor groups can enjoy the fruits of e-commerce development and effectively enhance their endogenous motivation. Moreover, through practical research, we find that all of farmers, village secretaries and agriculture-related e-commerce enterprises etc., recognize the core position of cooperatives in the implementation of e-commerce poverty alleviation.

**FIGURE 3:**
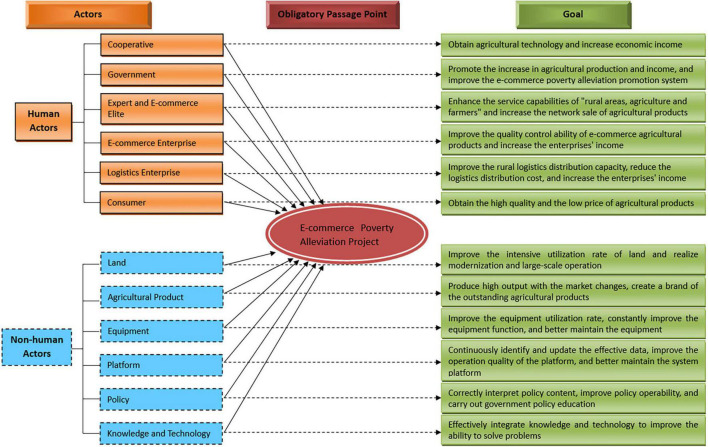
Problematization and obligatory passage point (OPP) of EPAAN.

In the process of e-commerce poverty alleviation, cooperatives propose the general goal of e-commerce poverty alleviation interest alliance based on their own interest demands, which is “believe that e-commerce poverty alleviation can promote industrial development in poverty-stricken areas, improve people’s living standards, and help people realize a new situation of getting rid of poverty and becoming rich.” Simultaneously, in order to achieve the respective goals of all actors, it is necessary to set up OPP to achieve the overall goal based on the interests of cooperatives. It is the “e-commerce poverty alleviation project.” The OPP is a prerequisite for the consolidation of interest alliances among various heterogeneous actors. It specifies the subject’s objectives for each actor, and it is as shown in Figure 3. Obstacle elimination and goal realization of all actors can enter the next stage of network construction only after passing through OPP.

#### Interessement

Interessement is a means to attract and stabilize the interest alliance relationship between core actors and other actors. It aims to encourage various actors to play the new roles assigned to them by core actors in the process of e-commerce poverty alleviation interest alliance, and clarify their own new roles orientation. Moreover, the ultimate interest of each actor is obtained by the interaction between itself and other actors, so as to realize the interaction and win-win of heterogeneous actors in the network interest alliance. In the process of e-commerce poverty alleviation, non-human actors and human actors with cooperatives as the main body, will encounter some obstacles in the development process due to their different positions. And these obstacles will limit their development. Therefore, it is necessary to clarify the obstacles and interests of all actors in the actor network, so as to continue to maximize the function of heterogeneous actors in the interest alliance. At present, according to the analysis results of survey data and combined with existing literature research, there are some obstacles and interests involved by the main actors in the EPAAN. They are as follows.

(1) Farmer cooperative. The main obstacles of farmer cooperatives are the lack of sufficient understanding of e-commerce poverty alleviation policies and the failure to give full play to the policy effects. Cooperatives lack the mastery of e-commerce operation technology and the flexible use of rural e-commerce. They lack of funds for development of agricultural product e-commerce and land for agricultural product production. The benefits of farmer cooperatives are to attract more farmers, expand the team of cooperative members, provide technical guidance to farmers, obtain e-commerce operation technology, obtain additional funds and land support, and improve the income and reputation of the cooperative, etc.

(2) Government. The main obstacles of the government are limited e-commerce technical support, incomplete e-commerce poverty alleviation promotion system, inaccurate grasp of e-commerce needs of grass-roots poor farmers and cooperatives, and low targeting of e-commerce poverty alleviation targets. The main benefits obtained by the government are to promote agricultural production and increase income, accurately identify poor farmers, greatly reduce the number of poor people, formulate policies to promote the development of cooperative e-commerce poverty alleviation, continuously improve the cooperative e-commerce poverty alleviation system, and achieve targeted poverty alleviation.

(3) Agriculture-related e-commerce enterprise. Agriculture-related e-commerce enterprises lack control of the source quality of agricultural products, lack the tightness of the upstream supply chain of agricultural products, lack in-depth understanding of the attributes of agricultural products, and lack the supply of agricultural products. The benefits obtained by agriculture-related e-commerce enterprises include effectively ensuring the stability and quality of the supply of agricultural products, improving the repurchase rate of agricultural products, increasing enterprise income, and doing a good job in quality control, etc.

(4) Logistics enterprise. The main obstacles for logistics enterprises in remote areas are imperfect infrastructure, few logistics distribution centers, weak logistics system, etc. For example, some fresh agricultural products are prone to corrode and damage. Hence, it needs to be distributed with cold chain vehicles, the logistics cost will be high. The main benefits obtained by logistics enterprises are to reduce logistics costs because of the increase in sales of e-commerce agricultural products, gradually improve logistics infrastructure, increase logistics distribution centers, etc. In addition, it will accelerate rural road construction and road upgrading, implement the express delivery project to the countryside, improve rural logistics distribution capacity, and optimize the rural logistics distribution mode.

(5) Expert and e-commerce elite. The main obstacles for experts and e-commerce elites are the lack of in-depth research at the rural grass-roots level, the lack of a comprehensive understanding of rural areas, agriculture and farmers, and the ineffectiveness of implementing e-commerce for poverty alleviation. The benefits obtained by experts and e-commerce elites are mainly to establish a high-level professional assistance team, improve the service capacity of “rural areas, agriculture and farmers,” and improve the online sales of agricultural products.

(6) Farmer. The main obstacles for farmers are the backward planting technologies, small planting scales and low agricultural income. The benefits that farmers receive are to access to the latest agricultural technology and increase in personal income.

(7) Consumer. The main obstacles for consumers are the limited ways to purchase regional characteristic agricultural products, and the limited choice of diverse agricultural products. The main benefits for consumers are to broaden the way to buy agricultural products, and enjoy a variety of special agricultural products with high quality and low price.

(8) Land. The main obstacles for land are the low utilization rate of land, and the phenomenon of idleness and waste. The benefits of land are mainly to increase the intensive utilization of local land and promote the large-scale, intensive, and standardized use of land resources.

(9) Agricultural product. The obstacles of agricultural products are that agricultural products have some characteristics such as seasonality and regionality. They are many varieties but not excellent, and they are widely distributed and scattered. They lack of scale and brand, lack of quality control, lack of unified standards, etc. The benefits of agricultural products mainly include solving the problems of unsalable agricultural products in poor areas and difficult sales for farmers, and improving the online sales capacity of agricultural products. It can improve the quality, standards and brand added value of agricultural products, and realize the upward trend of e-commerce agricultural products.

(10) Equipment. There are some main obstacles for the equipment. For example, the equipment function is imperfect and limited. The equipment life has a certain period, and the maintenance cost is high. The benefits obtained by equipment mainly include the development of functional diversity and perfection of the equipment, improving the utilization rate of the equipment, better maintaining the equipment and prolonging the service life of the equipment.

(11) Platform. The obstacles for the platform are that effective data cannot be identified due to the precipitation of massive data. The operation of the platform is complex. The cost of manual operation and maintenance is much higher and more cumbersome. The platform system has many loopholes, so it will be dangerous sometimes. The benefits obtained by the platform mainly include facilitating the effective identification and updating of data, doing a good job in the maintenance of the platform system, improving the operation quality of the platform, etc.

(12) Policy. There are some main obstacles of the policy. For example, it cannot accurately understand the connotation of the policy, and it cannot accurately grasp the timeliness of the policy. Moreover, the way of policy communication is limited. The benefits obtained by the policy are mainly conducive to the correct interpretation of the content of the policy. It can effectively grasp the policy in real time, smooth the channels of policy communication and feedback, and improve the openness and transparency of the policy.

(13) Knowledge and technology. The obstacles of knowledge and technology are the lack of e-commerce operation knowledge and practice. It is the difficulty in mastering and applying advanced planting technology, and the difficulty in integrating multidisciplinary knowledge and technology. The benefits of knowledge and technology mainly include the growth of e-commerce professional knowledge, the practice and renewal of professional planting technology, the integration and renewal of multidisciplinary knowledge and technology, etc.

**FIGURE 4:**
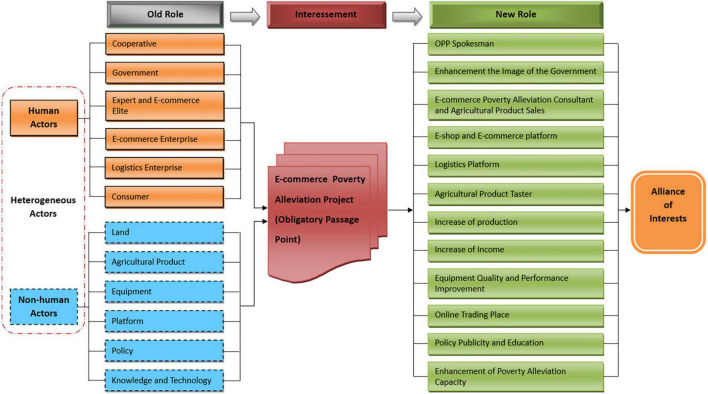
The interessement of EPAAN.

Through the presentation of obstacles and interests of various actors, it can redefine the new status and role of various actors in the EPAAN. All actors are recruited into the e-commerce poverty alleviation network alliance through interessement, and they play different roles in the network and play their own positive functions. It is as shown in Figure 4. If various actors have conflicts during the interessement phase, they must pass through OPP. They will continue to coordinate and cooperate to gradually form a stable interest alliance network around the OPP and their respective interest needs.

#### Enrolment and Mobilization

Enrolment is to find suitable actors for the EPAAN, accurately connect and remove the obstacles faced by various actors. It will attract actors to the network through a series of strategies and measures. It will ensure that all parties can take action, play their new roles and complete the corresponding tasks. The enrolment stage is closely related to the interessement stage. The interest correlation of various actors is the premise of enrolment. The recruited objects include not only human actors, but also non-human actors. They follow the arrangements of core actors, complete the assigned tasks and obtain the specified benefits. In the process of e-commerce poverty alleviation, cooperatives composed of farmers recruit the government based on the needs of poverty alleviation and industrial development. The government attaches importance to the construction of e-commerce poverty alleviation projects based on cooperatives, and provides a series of e-commerce poverty alleviation policy support. Moreover, the government will recruit a batch of agricultural experts and e-commerce elites to form a service group of “rural areas, agriculture and farmers,” which can go deep into the fields to help cooperatives and farmers solve various problems encountered in production and sales. Agriculture-related e-commerce enterprises and logistics enterprises are driven by economic interests to join the EPAAN and become members of the interest alliance. After human actors are recruited, non-human actors are also gradually recruited into the EPAAN such as land, agricultural products, knowledge and technology. After being recruited, the land becomes high-yield farmland, agricultural products become characteristic agricultural products. Knowledge and technology are recruited to improve the market competitiveness of cooperatives and farmers.

Mobilization means that the core actors fully mobilize the enthusiasm of all actors, execute their rights to all actors, and integrate them efficiently. In order to maximize benefits, they will work together, and finally form a stable and sustainable interest alliance. Callon believes that the actor network can be considered to establish completely only when the mobilization phase of the actor network interest alliance is done. In the EPAAN based on cooperatives, cooperatives have the strongest ability to mobilize farmers because their interests are most directly related. Moreover, cooperatives have the most obvious leading effect on farmers. The government has a stronger ability to mobilize cooperatives, but its ability to mobilize individual farmers is relatively weak. Other relevant organizations cooperate with cooperatives under the guidance of various government policies, actively participate in the e-commerce poverty alleviation system, and jointly achieve targeted poverty alleviation and become rich.

#### Dissent

Dissent means that the heterogeneous actors in the actor network hold different opinions on the distribution of interests. Through equal consultation and mutual coordination, it can eliminate differences and obstacles, and finally achieve the goal of joint cooperation among the actors. In the e-commerce poverty alleviation system, due to the large number of heterogeneous actors that are involved with diverse identities and complex demands, it is necessary to continuously coordinate the interests of all parties in order to promote the continuous update and stable development of EPAAN. In order to maintain the balance of interests among all members of the network alliance, it is necessary to deal with the dissent of various heterogeneous actors in the process of e-commerce poverty alleviation, such as the dominant position of cooperatives, the targeted identification of poor farmers, the targeted assistance of cooperatives to poor farmers, and the industrial structure and industrial layout for cooperatives, accurate interpretation of e-commerce poverty alleviation policies, sales methods of e-commerce agricultural products, brand effects of characteristic agricultural products, accurate implementation of e-commerce platforms and agriculture-related enterprises, etc.

**FIGURE 5:**
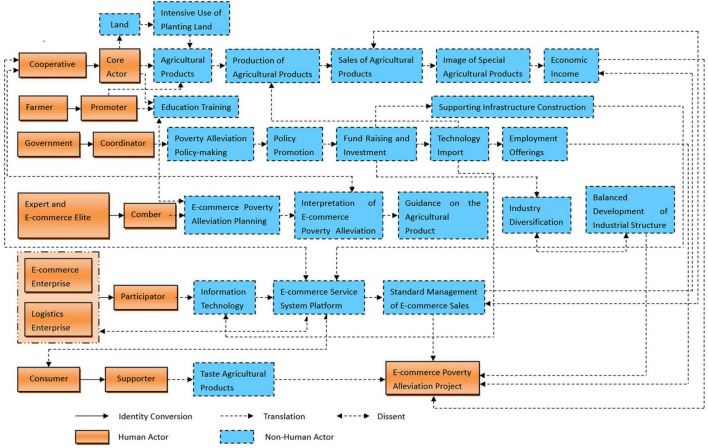
The construction of EPAAN system.

As the five stages in the translation process, problematization, interessement, enrolment, mobilization and dissent demonstrate the basic path for the formation and development of EPAAN. Although they can randomly participate in the network construction process, their mutual communication and efficient collaboration are the key points for the successful construction of the network. Based on the ANT, this paper analyzes the translation process of EPAAN, and finds that the translation process is based on the core actors to clarify the OPP jointly determined by each actor to achieve their own goals, so as to realize their benefit correlation among all actors. When human actors and non-human actors are recruited to become members of the interest alliance, it is necessary to ensure the balance of the interests of all actors, make all actors satisfied and play their new roles, and constantly coordinate and handle the interest conflicts of all parties. So that all actors with different interest orientations can establish a solid relationship with each other, actively participate in network alliance and play their respective roles. Through the translation process, the actors will transform into different identities. For example, the cooperative formed by farmers spontaneously becomes the core actor of the network alliance and the spokesman of the interest alliance. The farmers become the promoters of the network alliance, and the government becomes the coordinator of the network alliance. Experts and e-commerce elites become the combers of the network alliance, agriculture-related e-commerce enterprises and logistics enterprises become participators in the network alliance, and consumers become the supporters of the network alliance. With the changes in the interests of members, there are new actors to continue to enter or the original actors exit from the network, which will lead to the continuous renewal of the structure of EPAAN. ANT requires researchers to pay attention to the changes of interest needs of network alliance members, timely sort out the interest demands of various actors involved in e-commerce poverty alleviation, and gradually form a stable and orderly EPAAN through continuous dynamic translation. It is as shown in Figure 5.

## Discussion

### The Core Actor in the E-Commerce Poverty Alleviation Actor Network Is Farmer Cooperative

The ability poverty theory (Sen, 2000) enlightens us that the primary cause of farmers’ poverty is that farmers have poor feasible ability, especially the individualization and decentralization of farmers lead to the low degree of farmers’ organization. Therefore, the fundamental way to effectively solve the problem of farmers’ poverty is to improve the organizational level of farmers, and then enhance the feasible ability of farmers. The organization of farmers can resolve the contradiction between “small production” and “big market,” strengthen farmers’ bargaining power in the market, and reduce transaction costs, thereby increasing farmers’ economic income (Sivramkrishna and Jyotishi, 2008). Therefore, with the great advantages of cooperatives in improving the degree of farmers’ organization, it is determined that farmer cooperatives are the core actors in the EPAAN.

Our findings further suggest that the main task of cooperatives is to attract other actors to enter the EPAAN and work hard for the common goal, when cooperatives play the role of core actors. Hence, cooperatives should do the following jobs. Firstly, it is necessary to accurately identify poor farmers, guide them and mobilize their subject consciousness. Through stimulating their endogenous motivation, it can provide targeted assistance to them, and make them realize the importance of relying on organizational strength. Secondly, with the support of government policy, it can increase various training courses for farmers and members to study on e-commerce knowledge and production technology. Under the guidance of agricultural experts and e-commerce elites, it can continuously improve their skills. Thirdly, it should make an overall plan for e-commerce sales of characteristic agricultural products, and formulate relevant regulations to ensure the online sales of agricultural products. Through integrating the resources of various actors, it can help agricultural products enter the city for sale. Fourthly, it should increase capital investment and coordinate with various actors, especially agriculture-related e-commerce enterprises and logistics enterprises. Then, it should formulate enforceable systems and measures combined with the interest demands of various actors, so as to ensure the successful implementation of the sales process of e-commerce agricultural products, and finally it can form a stable e-commerce poverty alleviation actor network alliance.

### The Obligatory Passage Point in the E-Commerce Poverty Alleviation Actor Network Is Implementation of the E-Commerce Poverty Alleviation Project

Obligatory Passage Point (OPP) is an action plan that can be recognized and accepted by all actors in the network. Our results reveal that the implementation of e-commerce poverty alleviation project as an OPP is indeed conducive to highly unifies the thoughts and actions of all actors in the EPAAN. It can be unanimously recognized by all actors in the EPAAN, and effectively solve the problems faced by all actors, so as to achieve their respective goals. There are the main actors in the network, such as cooperatives, farmers, governments, agriculture-related e-commerce enterprises, logistics enterprises, agricultural experts, and e-commerce elites, etc. Focusing on the common goal of getting rid of poverty and promoting the development of local industries, they rely on e-commerce to improve their own initiative to participate in the EPAAN, and change the traditional sales mode of agricultural products in poor areas. They make use of internet infrastructure and cooperation platforms to integrate resources from all parties, expand sales channels of agricultural products, and reduce transaction costs and risks. Through e-commerce poverty alleviation, it can turn high-quality resources into wealth in poor areas, and improve the economic level of poor farmers. It can effectively improve the opportunities for poor farmers in cooperatives to participate in market competition. Moreover, it will help cooperatives promote the overall development of local industries and rural areas.

### The Emphases in the E-Commerce Poverty Alleviation Actor Network Are Multi-Party Cooperation and Joint Development of Rural E-Commerce

In November 2016, China incorporated e-commerce poverty alleviation into the overall deployment and work system for poverty alleviation. Moreover, various regions continued to explore the implementation of rural e-commerce applied to e-commerce poverty alleviation. In addition, agricultural products supported from e-commerce poverty alleviation had always achieved good sales results in recent years. In 2020, the total online retail sales of 832 national poverty-stricken counties was RMB 301.45 billion in China, which was an increase of 26.0% over the same period last year. Among them, agricultural product e-commerce continued to grow rapidly. Furthermore, the agricultural product online retail sales of national poverty-stricken counties was RMB 40.66 billion in 2020, which was an increase of 43.5% over the same period last year (The State Council of the People’s Republic of China, 2021). Meanwhile, the total number of online merchants in national poverty-stricken counties had reached 3.065 million by the end of 2020, which was an increase of 366,000 stores or 13.7% over 2019 (The State Council of the People’s Republic of China, 2021). Our finding not only prove that more and more farmers like to make online sales of agricultural products, but also demonstrate that rural e-commerce has become an effective strategy for e-commerce poverty alleviation to achieve targeted poverty alleviation. It plays an important role in promoting the development of industries in impoverished areas and driving the poor people out of poverty to become rich.

At present, e-commerce poverty alleviation has generated remarkable economic benefits and significant social impact. People from all walks of life are paying more and more attention to the role of rural e-commerce in anti-poverty, and they have reached a consensus on its role in poverty reduction. Therefore, how to develop rural e-commerce so that all the actors in the EPAAN can benefit and form a good cooperative relationship. It is necessary to deeply explore the factors that affect the in-depth cooperation of various actors and eliminate the obstacles as much as possible. Our research further confirms that the development of rural e-commerce is very helpful for poverty alleviation in poor areas. Through the development of rural e-commerce, all actors are allowed to establish a long-term collaborative mechanism. It needs to fully meet the requirements of participants by integrating resources of all parties and giving full play to the functions of all parties. For example, with the help of government departments, it can vigorously develop transportation facilities and communication infrastructure in remote rural areas, and open up the circulation channels of agricultural products between rural and urban areas. Furthermore, the government departments can provide various experts and e-commerce trainers for poor rural areas, and actively attract and cultivate rural e-commerce talents by different policies. For instance, with the help of cooperatives, agricultural-related e-commerce enterprises and logistics enterprises, it can promote the industrialization, scale and brand of agriculture. When actors reach a consensus, develop together and establish the sustainable EPAAN, they can really drive poor farmers to get rich and provide better opportunities and benefits from rural e-commerce to the utmost.

### The Difficult Point in the E-Commerce Poverty Alleviation Actor Network Is the Deep Integration of E-Commerce and Poverty Alleviation to Achieve Continuous Poverty Alleviation Without Returning to Poverty

The ultimate goal of e-commerce poverty alleviation based on cooperatives is to accurately identify and assist poor farmers under the help of cooperatives. Moreover, cooperatives as a carrier can improve poor farmers’ awareness on e-commerce, improve their vocational skills, and cultivate their ability to get rid of poverty. Our results offer that the deep integration of e-commerce and poverty alleviation projects can greatly promote the effectiveness of e-commerce poverty alleviation. Through the new format of e-commerce, it will deeply carry out the e-commerce poverty alleviation project. It will continue to maintain the endogenous growth of e-commerce, which has the hematopoietic function for poverty alleviation. It needs that all actors are able to identify their roles at different stages, cooperate with each other, and play a role at the right moment. Our results also paint three stages on the integration of e-commerce and poverty alleviation. In the first stage, e-commerce will be introduced to establish an upward sale channel of agricultural products to help the poor. It will attract more and more poor households through cooperatives, integrate local characteristic resources, and develop agricultural and sideline products industries which have market prospects. It can make poor farmers to initially enjoy the dividends brought by e-commerce. In the second stage, it will integrate resources from all walks of life to help the rapid development of agricultural product e-commerce. Government departments have issued various supporting policies to create a good development environment for cooperatives, agriculture-related e-commerce enterprises, logistics enterprises, etc. Agriculture-related e-commerce enterprises, logistics enterprises, experts and e-commerce elites will increase their support and cooperation to provide strong guarantees for cooperatives and farmers to develop characteristic agricultural products brands in the field of e-commerce. Simultaneously, consumers, agricultural products and equipment are all essential actors, and they will also play a role at key points. In the third stage, it will continuously improve the e-commerce poverty alleviation ecosystem and better serve poor farmers and industries. Through the implementation of the e-commerce poverty alleviation project, cooperatives should mobilize all actors to actively participate in the battle against poverty, and perfect the rural e-commerce service system that cooperates with all parties. In order to make the quality of e-commerce agricultural products meet the market requirements, it is necessary to implement the standardized, large-scale and brand management for e-commerce agricultural products to form industrial agglomeration and scale effect. It will achieve the solid poverty alleviation without returning to poverty, so as to help solve the problem of rural poverty.

## Conclusion

Poverty eradication is one of the difficult problems that need to be solved urgently in today’s society. Although the actual situation of countries around the world is different, they all actively adopt various methods to try to solve this problem. With the development of information and communication technology, especially the emergence of e-commerce, people have high hopes for the application of e-commerce to solve poverty. Scholars are actively studying it. At present, there are still few literatures on e-commerce poverty alleviation based on theory. Based on ANT, this paper proposes that when studying cooperatives in poor areas to drive farmers out of poverty, with the help of e-commerce, we should explore its formation mechanism and operation process from the aspects of actors, actor networks, obligatory passage point (OPP) and translation. Taking the poor counties in Zhejiang Province as an example, this paper puts forward the EPAAN model based on cooperatives.

From our research model, it can be found that the translation process of EPAAN based on cooperatives includes problematization, interessement, enrolment, mobilization and dissent. Our research results emphasize the role of the translation process of e-commerce poverty alleviation in the formation of a solid interest alliance network. Meanwhile, the research results not only emphasize the role of cooperatives in the entire e-commerce poverty alleviation ecosystem and ensuring the increase of farmers’ income and sustainable growth in industrial development, but also emphasize that all actors involved in the network have an equal status. The process of eliminating absolute poverty through e-commerce in poor counties in Zhejiang Province shows that the implementation of e-commerce poverty alleviation project based on cooperatives is a huge and complex system project. Once the interest alliance network of e-commerce poverty alleviation actors based on cooperatives is established, it can organize and develop itself to help more farmers in poor areas get rid of poverty and contribute to poverty alleviation. Therefore, the use of e-commerce is not only of great significance to the economic development of agriculture-related industries in developing countries, but also plays an important role in poverty alleviation work in poor areas.

Finally, our research not only plays a positive demonstration effect on how cooperatives in poor areas in developing countries use e-commerce to solve poverty problems, but also contributes to the overall research of e-commerce poverty alleviation and ANT. In the context of e-commerce poverty alleviation, it is expected that in the future, with the rapid development of cooperatives, agriculture-related e-commerce enterprises, logistics enterprises, finance, and infrastructure, as well as the issue of related policies, more and more agricultural products will enter the city through e-commerce. As a result, it will be common for more and more farmers to reduce poverty. Of course, our research has certain limitations. We take Zhejiang Province of China as a case study. Therefore, we hope that it can further enrich and perfect our theory in future research. Moreover, the biggest challenge of poverty alleviation is how to consolidate the achievements of poverty alleviation, to ensure that the poor people do not return to poverty and really get rid of poverty, as well as how to focus on high-quality development and build common prosperity. These will be research hotspots in the future.

## Data Availability Statement

The original contributions presented in the study are included in the article/supplementary material, further inquiries can be directed to the corresponding author/s.

## Author Contributions

All authors contributed to the development of the manuscript including the data collection, data analysis, and the writing phase.

## Conflict of Interest

The authors declare that the research was conducted in the absence of any commercial or financial relationships that could be construed as a potential conflict of interest.

## Publisher’s Note

All claims expressed in this article are solely those of the authors and do not necessarily represent those of their affiliated organizations, or those of the publisher, the editors and the reviewers. Any product that may be evaluated in this article, or claim that may be made by its manufacturer, is not guaranteed or endorsed by the publisher.
